# Perceptions of HIV cure research among people living with HIV in Australia

**DOI:** 10.1371/journal.pone.0202647

**Published:** 2018-08-24

**Authors:** Jennifer Power, Andrew Westle, Gary W. Dowsett, Jayne Lucke, Joseph D. Tucker, Jeremy Sugarman, Sharon R. Lewin, Sophie Hill, Graham Brown, Jack Wallace, Jacqui Richmond

**Affiliations:** 1 Australian Research Centre in Sex, Health and Society, La Trobe University, Melbourne, Australia; 2 Centre for Social Research in Health, University of NSW, Sydney, Australia; 3 School of Public Health, The University of Queensland, Brisbane, Australia; 4 UNC Project-China, University of North Carolina, Guangzhou, China; 5 University of North Carolina at Chapel Hill, Chapel Hill, NC, United States of America; 6 Faculty of Infectious and Tropical Diseases, London School of Hygiene and Tropical Medicine, London, United Kingdom; 7 Berman Institute of Bioethics, Johns Hopkins University, Baltimore, MD, United States of America; 8 Department of Medicine, School of Medicine, Johns Hopkins University, Baltimore, MD, United States of America; 9 The Peter Doherty Institute for Infection and Immunity, University of Melbourne and Royal Melbourne Hospital, Melbourne, Australia; 10 Department of Infectious Diseases, Alfred Health and Monash University, Melbourne, Australia; 11 Centre for Health Communication and Participation, School of Psychology and Public Health, La Trobe University, Melbourne, Australia; 12 Department of General Practice, The University of Melbourne, Melbourne, Australia; University of Toronto, CANADA

## Abstract

Participation in HIV cure-related clinical trials that involve antiretroviral treatment (ART) interruption may pose substantial individual risks for people living with HIV (PLHIV) without any therapeutic benefit. As such, it is important that the views of PLHIV are considered in the design of HIV cure research trials. Examining the lived experience of PLHIV provides unique and valuable perspectives on the risks and benefits of HIV cure research. In this study, we interviewed 20 PLHIV in Australia about their knowledge and attitudes toward clinical HIV cure research and explored their views regarding participation in HIV cure clinical trials, including those that involve ART interruption. Data were analysed thematically, using both inductive and deductive coding techniques, to identity themes related to perceptions of HIV cure research and PLHIV’s assessment of the possible risks and benefits of trial participation. Study findings revealed interviewees were willing to consider participation in HIV cure research for social reasons, most notably the opportunity to help others. Concerns raised about ART interruption related to the social and emotional impact of viral rebound, including fear of onward HIV transmission and anxiety about losing control. These findings reveal the ways in which PLHIV perspectives deepen our understanding of HIV cure research, moving beyond a purely clinical assessment of risks and benefits in order to consider the social context.

## Introduction

Early phase clinical trials investigating new pathways toward a cure for HIV are underway worldwide [1–3]. This research towards an HIV cure is aimed at identifying processes that will cure HIV or lead to long-term remission, allowing people living with HIV (PLHIV) to remain healthy without the need for ongoing antiretroviral treatment (ART) [4]. Clinical HIV cure research inevitably requires participation of PLHIV. This raises ethical concerns as most PLHIV who have consistent access to ART are healthy adults for whom HIV cure research trial will present risks, while offering no therapeutic benefits [5]. Also of concern are the risks associated with analytical ART interruption, which is likely to be part of most clinical HIV cure research trials. Analytical ART interruption involves suspension of ART for a period of weeks or months so researchers can monitor the length of time HIV remains supressed following a potentially therapeutic intervention [6]. Yet, ART interruption may contribute to resistance to some ART regimens, reduced CD4 cell count, or an increased viral load–all of which may lead to negative health consequences [7–14]. For these reasons, suspension of ART for any length of time runs counter to current HIV treatment guidelines [5,15].

There is a growing body of research that seeks to understand how these risks are perceived by PLHIV and affected communities [9,10,16–18]. The general aim of these studies is to determine the acceptability of HIV cure research and ART interruption among PLHIV who are potential participants in research trials. This body of research also builds on a history of scientific and medical research in which the ‘lived experiences’ of PLHIV have been considered alongside scientific research in the development of treatments for HIV [19,20].

In the late 1980s and early 1990s, HIV activists in the USA and other Western countries campaigned to have greater involvement in the design and oversight of clinical trials related to HIV treatment. This occurred at a time when there were very limited treatment options for HIV infection and PLHIV were facing almost certain death [21]. That concern created an urgent imperative for PLHIV activists to push for enhanced access to new HIV treatments, including access to treatments that were still experimental. Activists questioned the ethics and clinical trial procedures that delayed access to treatments for some people. They also claimed the right for PLHIV to determine the level of risk they were willing to accept as part of a trial, given the absence of safe and effective treatment options. This demanded greater consideration of the perspectives of PLHIV in scientific and clinical decision-making [19,22,23].

Consequently, the social history of HIV is characterised by a uniquely close working relationship between activists, researchers and clinicians [19]. In this relationship, PLHIV activists are positioned as informed spokespeople for potential trial participants. They also lay claim to a unique perspective in clinical research, as they are the people most affected by the processes and outcomes of such research [22]. In this context, the engagement of PLHIV in HIV-related research is not just about whether or not potential trial participants find HIV cure research trials acceptable, but also about recognising that PLHIV are positioned to bring a critical perspective to clinical research that may not be immediately apparent to clinicians, scientists or other stakeholders such as funders or policy-makers. The risks and benefits of clinical trials and the effectiveness of HIV treatment are not matters for scientific or clinical judgement alone. These issues can only be fully understood in the context of the lives of people who will be participating in trials, are directly affected by the outcomes of trials, or are using HIV treatment. Therefore, attention to the lived reality of PLHIV needs to be present at all levels of research [24].

HIV cure research sits in a very different category from the early HIV treatment trials in which participants were often willing to accept significant levels of risk and uncertainty as a result of the absence of any effective treatment for HIV infection [21]. Contemporary HIV cure research presents an inverted scenario wherein participants may be asked to risk their health when there is no chance of clinical benefit for them. PLHIV may also be asked to participate in HIV cure trials when the eventual outcome of such research is still uncertain. Trials are in their early stages and there is no guarantee that they will lead to therapies that are more acceptable or effective for PLHIV than current forms of ART [25]. The complexity of this scenario means that considering the lived reality of PLHIV in discussions on the ethics and processes of clinical and scientific research will be essential to advancing HIV cure science.

Recent surveys of PLHIV have shown that, despite the risks associated with ART interruption, there is a high level of willingness among PLHIV to consider participation in HIV cure-related clinical trials [7,10,17]. These studies suggest that motivation to volunteer for such trials is likely to be associated by perceived social benefits such as helping future generations or advancing science, and/or personal benefits such as gaining insight into their health [7,17,26]. These findings are supported by qualitative studies that point to altruism as a key motivation for PLHIV to participate in trials [16,27,28]. This research demonstrates the importance of exploring the meaning of risks and benefits of HIV cure trials through the lens of lived experiences of HIV infection. For example, Dubé and colleagues [16] conducted interviews about perceptions of risk and benefit in HIV cure research with a range of stakeholders including PLHIV, bioethicists and policy-makers, finding conflicting perspectives on risk and benefit. Drawing from objective measures of health, bioethicists and policy-makers viewed PLHIV as ‘otherwise healthy’ individuals whose health would be at risk in trial participation. In contrast, PLHIV experienced their health as vulnerable. Despite viral suppression and current good health, they still thought of themselves as at risk of poor long-term health and being exposed to other life challenges. As such, their perception of risk associated with trial participation was lower than that of bioethicists and clinicians.

Two reports discussing the motivations to participate in cure research among PLHIV who have already participated in HIV cure research point, similarly, to the value of exploring the motivation, risks and benefits of HIV cure research participation through the lenses of PLHIV [21,28]. Specifically, Henderson and colleagues found that PLHIV valued the sense of belonging and being part of a community of volunteers that came from trial participation [28]. Additionally, Evans, in his informal discussions with PLHIV who had participated in HIV cure trials, found that trial participation provided PLHIV with an opportunity to overcome negative experiences associated with their HIV diagnosis by being part of something that had potential to generate positive outcomes for others [21]. These social and emotional outcomes for PLHIV are not easily measurable; yet, not accounting for them may exclude important factors that influence PLHIV’s assessment of the risks and benefits of trial participation. As Evans (2017) noted: “While a general sense of satisfaction from altruistic intent may not be a sufficient counterbalance to the kinds of risks involved in some cure studies, it is important that we do not overlook a more profound and tangible benefit that some report to have acquired as a result of study participation.”

Building on this body of work, this article reports findings from a qualitative study with Australian PLHIV that examined the ways in which they perceived the risks and benefits of participation in HIV cure trials. We began from the perspective that PLHIV’s perceptions of HIV cure research–and the risks and benefits of trials–are likely to relate to their personal experiences of being diagnosed and living with HIV. We expand on previous research by exploring PLHIV’s attitudes toward HIV cure research in general and the potential for a cure to become available, as well as perspectives on ART interruption. The specific aim of this paper is to explore perceptions of, and attitudes toward, HIV cure research among PLHIV, including attitudes toward participation in clinical trials and the acceptability of risks associated with trial participation.

The Australian context provides a useful opportunity to conduct this research given that Australia was one of the first countries to implement intervention studies toward an HIV cure. HIV cure research also received considerable media attention in Australia during the International AIDS Conference hosted in Melbourne in 2014, and there is heightened awareness of HIV cure research among Australian PLHIV as a result [29].

## Materials and methods

Ethics approval for this study was granted by the La Trobe University College of Science, Health and Engineering Human Ethics Committee (approval number S15-152)

Qualitative methods were used for this study as our intention was to gain a detailed and nuanced understanding of concerns and issues regarding motivation to participate in HIV cure research among PLHIV. We adopted mixed theoretical models of qualitative research, incorporating deductive and inductive approaches to data collection and analysis [30,31]. Drawing from previous research, as described above in the Introduction, we had pre-existing ideas about factors that may influence participants’ motivation to participate in HIV cure trials which we wanted to explore. This included the likelihood that participants’ willingness to participate in HIV cure research might be linked to their level of optimism about a cure becoming available, their commitment to altruism and their attitudes toward risks associated with ATI interruption. Interview questions were directed toward these issues and, while we did not develop a codebook [32], we adopted a deductive approach to analysis, intentionally looking for text within the data that revealed a relationship between participants’ willingness to participate in trials and these issues. This is described further in the methods section below. We were also interested in participants’ narratives and attitudes toward participation in HIV cure trials more broadly–and were aware that our pre-existing ideas or hypotheses might not capture all relevant issues or themes. As such, we adopted an inductive approach that drew on grounded theory methods [33] in which we incorporated open questions into the interviews and utilised a data-driven, inductive approach to thematic analysis of data [34]. This is also described in more detail below.

### Recruitment and data collection

Individual in-depth interviews were conducted in 2016 with 20 Australian PLHIV aged over 18 years of age. The study relied on convenience sampling [35]. Interviewees were recruited through advertising distributed by HIV community organisations, HIV clinics and relevant online social networks. The advertising invited PLHIV to contact researchers if they were willing to participate in a one-hour interview.

The interviews were conducted either via telephone or face to face. Consent to participate was obtained prior to the interview via a written consent form and confirmed orally at the commencement of the interview.

Two researchers (AW and JP) conducted the interviews. Interviews were semi-structured and followed an interview guide (Table 1). Starting with the questions listed in Table 1, interviewers used prompts or probing statements (such as ‘tell me more’) to encourage interviewees to continue speaking or explain their answers. In addition, when interviewees raised issues or topics beyond the set questions, they were encouraged to explore this and say more [36].

**Table 1:** Interview guide.

General questions	When you were coming in to do this interview today, was there anything you were thinking or wondering about in relation to the topic of the interview that you can share with me?	
	Do you think that a ‘cure’ for HIV will become available in your lifetime? (Why/why not?)	
	Have you ever deliberately searched for information about HIV cure? (eg. Googled, asked doctors?) Can you tell me more about why you were searching?	
	Can you tell me what you know, if anything, about recent research aimed at developing an HIV Cure? Do you recall where you found this information? How do you feel about this recent research? How do you think other PLHIV feel about this research?	
	Are there circumstances in which you would consider participating in a clinical trial relating to HIV cure research? (Why/why not?)	
	What do you imagine this type of clinical trial might involve?	
	Do you imagine such a trial might involve risks to your health? What sort of risks? What level of risk would you be willing to take?	
	What would have to happen for you to consider yourself ‘cured’ of HIV? (eg. No meds? Not able to transmit HIV? Know definitely that HIV will never come back?)	
	How do you feel about the following cure scenarios:.	
		a. You no longer need to take HIV medication, and you cannot pass the virus on to others, but you could potentially get HIV again (you have no natural immunity).
		b. You still have HIV but you do not need to take daily medication to maintain viral suppression.
		c. Your viral load is undetectable and you no longer need medication to maintain viral suppression, but doctors are unsure if your HIV will rebound. You will need regular blood tests to monitor viral load for at least the next 5–10 years
	If a cure for HIV were to become available, how would it change your life?	
Hypothetical scenarios posed Interpretation of questions (interviewees were asked to tell us what they perceived to be the main information each question was seeking to elicit)	A clinical trial requires you to go off your antiretroviral medication which means your viral load may be unpredictable for up to one year. The trial is unlikely to provide any personal benefit to you, but may help develop scientific understanding that could eventually lead to a ‘cure’ for HIV. Would you be willing to participate in this trial? Why/why not? What, if any, are your major concerns?	
	If you had the opportunity to participate in an HIV cure-related clinical trial beginning tomorrow, how willing would you be to participate? (Not at all willing; somewhat unwilling; somewhat willing; very willing).	
	A clinical trial will involve taking extra medication on top of your antiretroviral medication for a period of two weeks. The impact of this new medication on your viral load is unknown and there may be some side effects. Would you be willing to participate in this trial? Why/why not? What, if any, would be your major concerns?	
If you indicated you are willing, to what extent would the following affect your willingness to participate? (Decrease willingness, no effect on willingness, increase willingness)	If participation would increase scientific understanding of a potential HIV cure but bring no benefit to your health	
	If participation would help future generations find a cure for HIV but bring no benefit to your own health	
	If participation would allow you to go off medication for a short period and still maintain viral suppression	
	If participation would give you access to specialist HIV medical care as part of the trial	
	If you were guaranteed access to medical care for the rest of your life if participation had a negative effect on your health	
	If participation may result in your viral load increasing or being less predictable for up to one year	
	If participation meant you may develop resistance to your current antiretroviral combination	
	If participation would make you more susceptible to disease and illness	
	If participation would require tissue biopsies or other invasive procedures	
	If participation required weekly visits to a medical clinic for several months	

Interviewees were asked a range of questions on their willingness to participate in HIV cure research based on their general knowledge of, and assumptions about, what participation might involve. The intent of these questions was to determine the interviewees’ baseline knowledge of HIV cure research, explore any assumptions they held, assess their senses of optimism about a cure for HIV becoming available, and explore their general thoughts about participating in clinical research related to HIV cure. After this, interviewers read out short vignettes (Table 1) that posed hypothetical scenarios relating to participation in HIV cure clinical trials in which the risks and benefits of participation were described. Interviewees were asked to comment on whether they would participate in an HIV cure clinical trial in the circumstances presented. Posing these scenarios was a strategy to encourage interviewees to reflect on possible points of uncertainty or contradiction in their thoughts on the risks and benefits of participation. These vignettes also allowed the introduction of questions about interviewees’ attitudes to ART interruption in the context of a various trial scenarios [37].

A second objective was to explore interviewees’ interpretations of the meaning and intention of a set of ‘closed’ questions (Table 1) on willingness to participate in HIV cure research trials. These questions were designed for use in a quantitative research instrument intended to be administered in a future study. Interviewees were asked to comment on how they interpreted these questions as a way to test and validate the questions. The data from this section of the interviews were originally not intended to form part of the dataset for this qualitative study. However, during the first two interviews we found that asking interviewees to comment on these questions encouraged them to think broadly about HIV cure research and raised topics that were not touched on in our original interview guide, including identifying some specific risks or activities that may be associated with clinical trial participation, such as invasive procedures or more frequent regular medical appointments. As such, we included responses to these interview questions in the dataset.

Following the interviews, interviewees were provided with the details of a reliable website where they could obtain more information on HIV cure research being conducted in Australia (http://hivcure.com.au/). Researchers made it clear to all interviewees, both before and after interviews, that this study was not connected to clinical research and that there is currently no cure for HIV or any evidence that a cure is likely to become available in the near future. We were concerned that people who were newly diagnosed with HIV might be more vulnerable, or more likely to find the interviews distressing, than people who had been living with HIV for many years. To address this, researchers took extra time to speak with newly diagnosed people prior to the interview in order to ascertain whether they had access to appropriate information about living with HIV, clinical care and support, and to ensure they understood that this study was not linked to clinical research or a possible cure for HIV.

### Data analysis

All interviews were transcribed verbatim. Two researchers (AW and JP) separately conducted a thematic analysis of the transcribed data using two approaches:

- Data were coded according to set of topics of interest, including the relationship between willingness to participate in HIV cure research and optimism about a cure becoming available, sense of altruism and concerns about health risks (particularly with respect to ART interruption). This approach drew on what Hsieh and Shannon (2005) describe as ‘directed content analysis’ in that this was a theoretically deductive data-analysis process in which the goal was to validate and extend our existing knowledge of the topic [38]. The researchers paid attention to places in the text where the abovementioned topics of interest were mentioned and intentionally considered what this revealed about each participant’s willingness to participate in HIV cure research.
- Inductive thematic analysis was undertaken to organise codes into broader themes and sub-themes and identify codes that sat outside the known topics of interest. This method was adopted as a way to identify unexpected themes, explore complexities and contradictions in the data (including contradictions within the narrative of each interviewee and between interviewees), and break the data into sub-themes. Researchers identified core themes by paying attention to emphasis in interviewees’ narrative and repetition in the interviews [39].

Once each researcher had completed an independent analysis, consistencies and discrepancies in the findings of each researcher’s analysis were identified through discussion and collaboratively writing notes and memos. This method of cross-checking consistency of coding between two researchers was intended to ensure rigour in the method of coding and confirm consistency in the interpretation of themes [40]. With respect to the inductive analysis, the process of discussion and collaborative writing facilitated detailed engagement with the data, as the researchers each went back to the data to work through inconsistencies in interpretations as well as to develop and enrich themes [41].

The characteristics of the 20 interviewees are listed in Table 2. In order to protect the privacy of interviewees, we have grouped information in the table and have not used pseudonyms or case numbers to attribute quotations presented in the results section.

**Table 2:** Participant characteristics.

	Age	Gender	Sexuality	Time since diagnosis	Willingness to participate in HIV Cure trials
1	30–40	Male	Gay/bisexual/non-heterosexual	Less than 12-months	Would consider participation in a trial if he assessed it as scientifically sound.
2	40–50	Male	Gay/bisexual/non-heterosexual	10–15 years	Would consider participation in a clinical trial.
3	40–50	Female	Heterosexual	15–20 years	Maintaining general health is of high importance due to caring responsibilities so would not participate in a trial on this basis.
4	40–50	Male	Gay/bisexual/non-heterosexual	10–15 years	Would consider trial participation although had other health issues which he feels would make him ineligible.
5	50–60	Male	Gay/bisexual/non-heterosexual	20–30 years	Would participate in a trial.
6	40–50	Male	Heterosexual	30–40 years	Would participate in a trial.
7	20–30	Male	Gay/bisexual/non-heterosexual	Less than 10 years	Very interested in HIV cure research intellectually and personally interested in contemplating the implications of HIV cure for his own life. Would participate in a trial.
8	40–50	Male	Heterosexual	Less than 10 years	Would participate in a trial.
9	60+	Male	Gay/bisexual/non-heterosexual	20–30 years	Some familiarity with HIV cure research as part of his employment in HIV sector. Would participate in a trial.
10	50–60	Male	Gay/bisexual/non-heterosexual	20–30 years	Would consider participating in a trial if impact on life and work was minimal.
11	40–50	Female	Heterosexual	Less than 12-months	Very interested in learning more about HIV cure research, came across advertisement for this study while searching for information about current research. Would participate in a trial.
12	60+	Male	Gay/bisexual/non-heterosexual	20–30 years	Would participate in a trial but not willing to suspend ART due to previous negative experience of ART break.
13	40–50	Male	Gay/bisexual/non-heterosexual	10–20 years	Been involved in previous clinical trials for HIV medication and would consider future participation in other HIV trials, including cure trials.
14	40–50	Male	Gay/bisexual/non-heterosexual	Less than 10 years	Would participate in a trial if it did not impact on health.
15	60+	Male	Gay/bisexual/non-heterosexual	10–20 years	Positive about potential for a cure after reading press reports so interested in hearing more. Would participate in a trial although concerned about health impact.
16	40–50	Male	Gay/bisexual/non-heterosexual	20–30 years	Would participate in a trial if clinic was in an accessible location.
17	50–60	Male	Gay/bisexual/non-heterosexual	20–30 years	Would participate in a clinical trial.
18	40–50	Male	Gay/bisexual/non-heterosexual	Less than 10 years	Previously been involved in HIV treatment trials and would consider participation in cure trials.
19	60+	Male	Gay/bisexual/non-heterosexual	20–30 years	Not sure if he would participate in any clinical trials due to age.
20	20–30	Male	Gay/bisexual/non-heterosexual	Less than 12-months	Would consider participation in a trial but interested in learning more.

### Participant characteristics

Eighteen men and two women participated in an interview. Sixteen of the men identified as ‘gay’ or as another non-heterosexual identity such as ‘pansexual’ or ‘men who have sex with men’. This is consistent with the pattern in the Australian HIV epidemic where, in 2016, less than 10% of people living with HIV were women (approximately 3,000 women in total) and close to 70% of HIV transmissions occurred through male-to-male sex [42]. Interviewees’ ages ranged from 23 to 64 years, and the length of time individuals had been living with HIV ranged from less than six months to 31 years. All interviewees spoke fluent English, although one participant indicated that English was a second language.

No interviewees had participated in HIV cure related clinical trials, although four had previously participated in HIV treatment trials. Ten interviewees described themselves as having some involvement with the HIV sector: five were employed by HIV community organisations; two had a clinical background in HIV; two were volunteers in HIV community organisations; and one had previously been involved in the HIV sector as a volunteer and activist but had little involvement in recent years.

## Results

The findings from this study reveal that interviewees’ perceptions of HIV cure research and their feelings on potential participation in cure trials (including associated risks) were grounded in their social and emotional experiences of living with HIV, rather than in an objective assessment of the status of cure science or medical information on it. Interviewees indicated they would be motivated to participate in HIV cure research for social reasons, namely to support other PLHIV. Similarly, the risks of ART interruption were interpreted by many interviewees in social rather than medical terms. We outline these findings in more detail below.

### Attitudes toward HIV cure research

We asked interviewees about their existing familiarity with HIV cure research, if they had ever searched for information about curing HIV, their general thoughts about the topic of HIV cure, and whether they believed a cure for HIV would become available in their lifetime. Responses to these questions revealed that interviewees’ engagement with information on HIV cure and their sense of optimism that a cure would become available in their lifetime were often grounded in their emotional experience of living with HIV rather than any factual understanding of the current status of HIV cure research. For example, several interviewees indicated that they were disengaged from information about HIV cure research–or chose not to think about whether a cure would become available–as a way to avoid disappointment. As one interviewee stated:

Certainly, in the first few years of diagnosis, every time I saw an article in the press about a potential cure for HIV I was on it and got very excited sort of thing. And when, some months later, when nothing came of it, I’d get disheartened, sort of thing, and address that with a counsellor who’d had many, many, many years of experience counselling HIV clients and, basically, helped me to let that go a little bit and said you are setting yourself up for that kind of disappointment.

Similarly, a few interviewees indicated that they had made an active decision not to engage too closely with information about HIV cure research or consider the possibility of a cure, because holding out hope for a cure made it more difficult for them to be optimistic about the day-to-day reality of living with HIV. As one interviewee stated:

I try to actively not think about whether a cure would be available in my lifetime. There was a long time in which I chose to believe there would not be a cure in my lifetime because I never wanted to wake up one day having the reason being to get out of bed be there might be a cure today.

Another interviewee said that it had been a deliberate choice to disengage from information about HIV cure, stating:

I decided many, many years ago that I wouldn’t hold out for a cure. So, I would just live my life.

Consistent with these quotations is general reluctance among interviewees even to engage with the possibility that a cure might be possible in their lifetime. One interviewee expressed this as a sense of losing heart over time, saying:

Every now and then I’ll attend a lecture or something, you know, from a professor or someone to say where we are at with HIV cure. I lost a lot of heart in the years that were lost looking for a vaccine, which I always thought was a stupid idea, from my perspective, because it wasn’t going to help me, and the many, many others that live with HIV. But I honestly don’t think people living with HIV [are] paramount in planning [responses to HIV] … I don’t think anyone really believes there's going to be a cure, I just don’t think that it, that is, yeah, no one talks to me about that.

Other interviewees expressed cynicism about the commercial motivation to develop a cure. Indeed, four interviewees directly stated that commercial interests in ART, as opposed to a cure, were a major reason why they did not feel optimistic that a cure would become available in their lifetime:

I don’t think we will find a cure in my lifetime, and I used to work in big pharma before I got HIV and pharma is about business, big business, it is not profitable to cure, it is profitable to suppress and to maintain, to make it liveable to live with the disease. And from that point, it's my general, I guess, impression that it's just not a good idea to fix HIV from a commercial point [of view].

[They] turned it into a chronic manageable illness, and that seems to be all that medicine wants to do about everything, you know. So yeah, so it's quite surprising to me that there's such currency with cure research.

### Willingness to participate in HIV cure research

Despite interviewees’ overall low level of awareness of information about HIV cure research and a general pessimism about a cure becoming available, almost all interviewees indicated they would be willing to consider participation in clinical trials related to HIV cure research (see Table 2). Even the interviewees quoted above, who were convinced the commercial success of ART would be a barrier to the advancement of cure science, were willing to consider trial participation. When asked why they would consider participation in a trial, one of these interviewees stated:

Absolutely. [Why so sure?] Because of the experience I’ve had with my brothers and sisters at the beginning of the epidemic, I just thought that, as a human being, that it's just a beautiful piece of altruism, and if it can bring cure to future generations, that’s a sacrifice I’m willing to make.

On one hand, this response seems inconsistent with these participants’ earlier convictions that a cure would be unlikely due to the profitability of ART. On the other hand, it is consistent with a core theme found in these data: that PLHIV’s interest in HIV cure research is connected to their social and emotional experience of living with HIV. Many interviewees were willing to participate in HIV cure clinical trials because they saw it as an opportunity to give something back to communities and to other PLHIV, something that they considered important to them or which offered emotional benefits. For example, some older interviewees saw clinical trial participation as an act of generosity they could gift to future generations. One interviewee even described this as a sense of obligation to young PLHIV:

For me, I feel that there's part of, it feels like an obligation, but not in a bad sense, I feel obliged to carry some of the load, not just for myself. You know … even though I don’t feel it at the moment, I’m usually incredibly resilient and I have very broad shoulders. And so, I’m willing to help carry a burden for others that can’t. and I suppose it's the same here with participation in clinical trials. You know, it's not just for benefit for me; it's the benefit to all, and for me that’s, it's just who I am. It's just the way I’ve been brought up. I’d actually say it’s part of my DNA, because I think that’s how my mum’s side of the family … it's this givingness. So for me to be involved and participate in a clinical trial is giving, it's a really, it's a positive balance in my ledger to do, yeah.

Another interviewee, aged over 50, saw trial participation as a way to support young PLHIV.

Turn yourself into a guinea pig for the good of the young gay guy. I’m always thinking about that baby [gay man], the one that’s just about to be diagnosed. Now he will never have to go through what we went through and that’s good. So you just make it all that little bit easier.

Young interviewees also saw trial participation as an opportunity to give back to the community that had helped them, or to make a difference for the future. One interviewee described this as being part of the ‘bigger picture’:

And it's also my way of giving something back, because I’ve, like, I’ve met some beautiful people through this journey in the last two and a half years and, yeah, it's just being part of the bigger picture I guess.

For another young and newly diagnosed interviewee, trial participation was seen as a way to respond to being diagnosed with HIV in an active way by being helpful and pushing research forward:

I just, you know, I’m disappointed that I have been contracted with [HIV] (sic), but I’ve sort of got this mindset whereby, you know, I’m just going to have to make the most of it and, like, if I can be helpful I’ll try to be. I don't know. Maybe it's for personal selfish reasons, you know, a cure would be wonderful. But, you know, I’d like to think it’s a bit more of an altruistic sense of, you know, I can do my duty, or see what I can do to help …

[*Later in the interview*] … I think, yes, for me personally now, maybe because I’m younger and I’m more risk taking, but I think I would [participate in a trial]. Because I don't know, like, I have this, sort of, feeling at the moment that I’m on antiretroviral medication, I’m at an undetectable level. But it's, sort of, like, I’m, sort of, [in] a position of stasis or, you know, stagnation that I’m not sort of helping. I want to help push things along, so I think I would, if that makes sense.

Only one interviewee referred to the possibility of becoming cured as a motivation to participate in a trial. Even for this interviewee, the desire to participate was linked to wanting greater involvement in research on a topic so ‘close to my heart’:

Well, I’d be, I’d like to think, like, it is something that’s very close to my heart, given that I’m living with it. And being part of that trial, it could potentially mean that I could be on the road of getting cured, 100%, before anyone else, but before it reaches the rest of the community.

For one other interviewee, the possibility of personal benefit was intertwined with altruism or being a good citizen:

Oh, I think there is a multitude of reasons for a guy in his … middle age … who has lived with HIV to participate [in a trial]. The main ones will be, I suppose, the possibility of a personal benefit in terms of, maybe, improving one’s personal–my own personal–immune system, and maybe reversing some of the damage done by HIV in my body. But then, I suppose, there’s also issues around doing it as a good corporate citizen. And I think the constraining things in that space will be how much of a risk would it be to my ongoing control of HIV.

### Willingness to accept risks in trial participation

The interviews included several questions regarding interviewees’ perception of the risks involved with HIV cure research trials and whether they would be willing to suspend ART for a short or long period of time. Overwhelmingly, interviewees were willing to consider some risk to their health associated with clinical trial participation, such as side-effects from medications, or a period in which their viral load became detectable. Interviewees placed limits on the risks they were willing to assume if it meant they could no longer work due to illness or if their CD4 count dropped significantly. One interviewee, who was a woman with family responsibilities, said:

[If] I were to go off my [HIV] medication then my body doesn’t cope. So there might be times of, maybe, without–without meaning to, there may be hospitalisations or ill–you know, gosh, if I get the flu for a couple of days my house falls into ruins even though I have a husband. Because I am the, I am part of the, the main person here, so–and I would imagine clinical trials, as you said, would take up time, as the question said, take up time and you’d have to be able to commit personal time and I don’t have that.

The risk of becoming unwell or not being able to work was noted, particularly by those who had experienced poor health or time out of the workforce due to HIV. One interviewee said:

Yeah, you bet, absolutely, yeah [I would participate in a trial]. But I’d need to know why, I’d need to–I definitely am not going to just show up on a blind trial and say here take these drugs–you know. I’ve struggled too hard. I’ve been through too much to just give it all away.

Emerging with this, however, was a sense of the social and emotional impact of suspending ART. For some interviewees, an increasing viral load was linked to feeling like they had lost control of HIV, which had potential to be distressing even if there were no health impact. This was expressed by interviewees in different ways. One interviewee stated: ‘So, to have the prospect of [viral suppression] being disturbed is frankly quite frightening’. This interviewee explained that viral suppression offered reassurance of good health and no risk of passing HIV on to others, such that ‘any deterioration from that situation is obviously something that’s really concerning’. Another interviewee explained that losing viral suppression and risking ART resistance would go against everything PLHIV are taught about HIV management:

[The trial] may result in your viral load increasing or being less predictable for up to a year? Mmm, going against the Holy Grail with that one. The one thing we’ve drummed into everyone is that low viral load is where we’ve got to be … If participation meant that you may develop resistance to your current antiviral combination. We may have just found a deal breaker for me … because resistance is the big issue that we face … So, you know, it's not a no, but resistance is a big thing for us, so we’ve got to be mindful of that. So that question conjures a whole bunch of stuff inside of me that would need some more questions and answers.

The possibility of having a detectable viral load did not mean interviewees rejected the possibility of participating in trials that involved ART interruption, but it did lead interviewees to reflect on what it meant for them, socially or emotionally, to have an undetectable viral load. One interviewee explained that viral suppression was associated with a particular status–‘undetectable’–and this is a marker of good health and low risk of transmission:

I think having an unpredictable viral load for one year is not particularly damaging to one’s body, especially if you're in a trial [and] it's being monitored quite closely. But I think a lot of people rely on their undetectable status, and to give that ‘up’ in the social sense . . . . I think this one would take consideration, I’m not sure it would deter me, but I think I would have to give it a lot more thought before going into the trial.

Another interviewee also raised concerns about what it would mean for sexual relationships (although the possibility of a partner taking pre-exposure prophylaxis was not discussed in the interview):

Because it would mean that you'd have to be very, very careful. Well, I’m not in a long-term relationship at the moment. But if I was at the time, I would have to double think it, or you'd have to, you know, there’d be an extra person in this decision-making process. And they’d say, you know, well, they would have to take more precautions and whatnot. And it’d probably lead to reduced sexual activity, but again that’s a hypothetical. I don't know exactly. It depends on who the hypothetical partner was and all that. But, I think, you know, from a purely personal point I would say yes.

## Discussion

PLHIV are the primary stakeholders in HIV cure research as they are the people who stand to benefit most from the outcomes of such research. They will also be asked to volunteer for successive clinical trials, including those that involve risks to their health. As such, the advancement of HIV cure science relies on being considered valuable and ethical by PLHIV, not only individually but as a community of stakeholders. Thus, there is a clear need to engage PLHIV in HIV cure research. Politically and ethically, it is also important that HIV cure research aligns with the United Nations’ principles of Greater and Meaningful Participation of PLHIV in research [43]. That said, involvement of PLHIV in HIV cure research is also about the importance of medical and scientific researchers considering a perspective on HIV cure research that is grounded in the personalised knowledge and experience of PLHIV, not just in clinical or scientific assessments of risks and benefits. The lived experiences of PLHIV have been highly valuable to so much medical, scientific and social research on HIV infection and AIDS [19] and promise to offer a similarly valuable perspective on cure research that may not be immediately obvious when considered only through a singularly ‘scientific’ lens. Moreover, clinical trials involving ART interruption involve known and measurable risks to the health of PLHIV [12]. Therefore, in the absence of known and measurable clinical benefits, it is only PLHIV themselves who can determine if these risks are worth taking.

This paper builds on existing work [16,21,28] to demonstrate the importance of considering the lived experiences of PLHIV in the advancement of HIV cure research. Previous research has shown that PLHIV may not assess the risks and benefits of participation in HIV cure clinical trials in terms of easily measurable impacts on health or viral suppression [16,28]. Instead, risk and benefit are often viewed through a subjective lens–including people’s sense of vulnerability to health problems [16], or the emotional benefits of helping others through being in a trial [21]. This study provides further support to these findings. Interviewees’ perspectives on HIV cure research were not just based on rational assessments of contemporary cure science, but also on their own personal strategies for managing hope and disappointment in relation to a possible cure for HIV. At a personal level, for many interviewees, their assessments of the risks in ART interruption were based on the emotional and social impact of ART interruption rather than the medical risks alone. Some interviewees even spoke of the potential distress of losing a sense of stability and control in the management of their HIV infection. Treatment adherence and monitoring of viral load are central to the clinical management of HIV, and viral suppression is an indication that HIV is ‘under control’, which may be psychologically important for PLHIV. This is evident in studies that show an association between increased viral load and higher levels of distress or depression among PLHIV, irrespective of the impact of viral load on physical health [44]. At a social level, one interviewee also clearly expressed concern about losing the status of ‘undetectable’ (viral load). Recent research has demonstrated the efficacy of ART-induced viral suppression in preventing onward transmission of HIV [45]. Such research has informed contemporary HIV anti-stigma campaigns, including the international ‘U = U (undetectable = untransmissible)’ slogan, which is a major international campaign that challenges HIV stigma by building awareness of the effectiveness of modern ART in mitigating the risk of HIV transmissibility [46]. There may be personal and social concerns for PLHIV associated with a detectable viral load–including stigma–that are not necessarily clinically apparent, but which affect perceptions of risks and benefit of participation in HIV cure research [47].

The findings presented in this article encourage consideration of the other potential benefits of participation in a HIV cure clinical trial from the personal and social perspectives of PLHIV. While most interviewees in this study adopted a pessimistic attitude to the possibility that they themselves might be cured of their HIV infection, most indicated they would consider participating in a clinical trial because they saw it as something they could do to help others. For many interviewees, this was grounded in their own experiences of other people helping them, and/or in their sense of PLHIV being a community. This finding is consistent with Evans’s argument that altruism may provide motivation for people to participate in a trial, but it may also be useful to reframe altruism as a benefit that people may derive from trial participation [21]. For example, trial participation may provide the opportunity to derive positive meaning from the experience of being diagnosed with HIV. This was the case for one interviewee for whom participating in research was a way to respond positively to their HIV diagnosis, which was otherwise challenging and difficult. Being helpful and being part of possible scientific advancement can be a strategy for re-orienting the meaning and experience of an HIV diagnosis. This finding adds weight to previous research that showed that recognising the lived experiences of PLHIV is important in advocating for the potential benefits of participation in HIV cure research, even where there is no expected therapeutic benefit [21,28]. The participation of PLHIV in research at its many levels–not just as enrolled subjects in trials–has been a cornerstone of the global response to the HIV pandemic. The term ‘altruism’ does not adequately capture the role that PLHIV have played in the HIV response because, just as it has done to previous and ongoing research on behaviour change, prevention, treatment and vaccines [19], the act of living with HIV itself will bring valuable understanding and depth to HIV cure research.

### Limitations

There are several limitations to this study. First, being based in Australia, interviewees all had access to publicly funded healthcare and subsidised ART. Seeking improved healthcare or treatment may be a factor motivating people to participate in clinical trials in settings where such healthcare is less readily available. The sample size for this study was small and interviewees were predominantly Caucasian Australians, the majority of whom identified as gay men or men who have sex with men. This is not uncommon in studies of PLHIV in Australia and broadly reflects the demographic characteristics of PLHIV in Australia [48]. However, the findings here may not reflect the views of people from migrant and non-English-speaking backgrounds or many women living with HIV. Finally, this study utilised hypothetical scenarios as a tool to discuss with interviewees’ potential risks associated with treatment interruption; future studies may benefit from asking interviewees about more specific, planned trials and/or study designs to determine acceptability. There is also only limited research to date that has sought to explore motivations for trial participation among people who have actually participated in HIV cure related clinical research [28,49]. Further research with trial participants would be valuable to explore the extent to which anticipated benefits equate with the reality of their trial experiences.

## Conclusions

Social research, such as this study, enables scientific researchers to understand how PLHIV perceive HIV cure research and provides insight into the risks and benefits of HIV cure-related clinical trial participation. This research is also one facet of community engagement processes that are essential for HIV research in general and HIV cure research in particular [10]. We believe that incorporating the perspectives of PLHIV into messages about HIV cure research and the design of trial protocols will improve HIV cure research. PLHIV provide a unique perspective on the risks and benefit of clinical trials that is grounded in the personal and social realities of living with HIV. There is a strong history in the HIV sector of PLHIV and community activists working closely with researchers, clinicians, policy-makers and funders [19,23]. Further advancing HIV cure science will depend on sustaining this important partnership. Key to the success of this partnership is recognizing that different forms of expertise and insight are necessary to understand the full value and impact of scientific research in its pursuit of a cure for HIV infection. Our study highlights the value of PLHIV perspectives as biomedical HIV cure research advances in global settings.
